# Chemotherapy and Targeted Therapy Strategies in Patients with Unresectable or Borderline Resectable Metastatic Colorectal Cancer: Evidence for a Lack of Focus on Resection Rates

**DOI:** 10.1245/s10434-023-14049-3

**Published:** 2023-08-30

**Authors:** Jan Zmuc, Jan Heil, Caroline Herfarth, Wolf O. Bechstein, Christine Koch, Jörg Trojan, Andreas A. Schnitzbauer

**Affiliations:** 1 Department of Surgical Oncology, Institute of Oncology Ljubljana, Ljubljana, Slovenia; 2 Department of General, Visceral and Transplant Surgery, Frankfurt University Hospital, Goethe-University, Frankfurt am Main, Germany; 3 Department of Medicine I, Frankfurt University Hospital, Goethe-University, Frankfurt am Main, Germany

## Abstract

**Background and aims:**

Chemotherapy (CTx) with targeted therapy (TT) have increased the overall response rate (ORR) and improved survival in unresectable or borderline resectable metastatic colorectal cancer (mCRC). However, the resection rate is an endpoint with often suboptimal expert involvement. The aim was to investigate whether the improvements in ORR have translated to improved resection rates (RR).

**Study design:**

A systematic literature search was performed using the PICO process.

**Statistical analysis:**

Odds ratios, and 95% confidence intervals (OR, 95% CI) were analyzed for ORR and RR using dichotomous values with the Mantel-Haenszel method. Progression-free survival (PFS) and overall survival (OS) were analyzed using the inverse-variance method and displayed as hazard ratios and 95% confidence intervals (HR, 95% CI).

**Results:**

The literature search returned 469 records. Sixteen articles with 5724 patients were selected for analysis. The qualitative analysis revealed low and moderate risk of bias endpoints. Higher ORR was observed with CTx + TT versus CTx only (OR: 0.62 [95% CI 0.45; 0.82], *p* = 0.002) and with triplet CTx + TT versus doublet CTx + TT (OR: 0.61 [95% CI 0.46; 0.81], *p* < 0.001). PFS and OS were improved by use of TT (HR: 0.68–0.84; *p* < 0.001 to 0.04). The overall RR was low (< 15%) and did not improve in the same way as the other endpoints.

**Conclusion:**

The ORR and survival rates in unresectable and borderline resectable mCRC were improved by modern CTx and TT that did not translate into higher RR, mostly due to the lack of expert involvement.

**Supplementary Information:**

The online version contains supplementary material available at 10.1245/s10434-023-14049-3.

Metastatic colorectal cancer (mCRC) is a major cause of morbidity and mortality. Patients with mCRC can be treated with surgery, ablative techniques, chemotherapy (CTx) and targeted therapy (TT) with anti-epidermal growth factor (EGFR) or anti-vascular endothelial growth factor (VEGF) drugs. Surgical removal of metastases is potentially curative; however, only a minority of patients with mCRC are initially suitable for resection and the concept then is instead to reach a prolonged chronic state of disease control improved by surgical and other interventional therapies such as ablation or radiation therapy.^1–4^ The latest data have shown the tremendous positive impact of surgical resections, leading to 5-year survival rates of 60% and more when compared with patients that were not resectable, who experienced 5-year survival rates of 30% and lower.^5^ Modern CTx and TT have increased the overall response rates (ORR) to systemic treatment of mCRC. Therefore, systemic treatment with CTx and TT can be used to try to convert initially oligometastatic unresectable mCRC to resectable disease.^4,6^ Most of the randomized controlled trials (RCT) published so far do not consider the metastasis resection rate (RR) as a well-defined endpoint supported by expert decision making, and have focused instead on ORR, progression-free survival (PFS), and overall survival (OS). This is a systematic literature search and meta-analysis to explore the relationship between ORR and metastasis RR in patients with initially unresectable or borderline resectable mCRC. The hypothesis was that the RR did not improve in the same way that ORR, OS, and PFS did with the use of modern CTx and TT.

## Methods

### Research Question

Do improvements in ORR with modern CTx and TT translate to higher metastasis RR in patients with upfront unresectable or borderline resectable mCRC?

### Literature Search Strategy

The following elements were employed in the literature search strategy:

- Population: Adult patients older than 18 years with mCRC.
- Intervention, Comparator: Use of different types of antineoplastic agents for systemic treatment of mCRC.
- Outcomes: ORR, RR, PFS, OS.
- Timing: 1 January 2000 to 1 January 2020.
- Study type: RCT.

A systematic literature search of the MEDLINE/Pubmed electronic database using the Medical Subject Headings (MeSH) “colorectal neoplasms,” “neoplasm metastasis,” “antineoplastic agents,” and “randomized controlled trial” was carried out to find appropriate articles. The results were limited to English articles published in the time period between 1 January 2000, and 1 January 2020. Additionally, the references of the chosen articles were screened to minimize the risk of missing relevant publications. For multiple publications reporting on the same patient population, the most recent publication was used.

### Inclusion and Exclusion Criteria

The following inclusion criteria were used to select the relevant articles for analysis:

- Included patients were adult humans older than 18 years with mCRC.
- Included patients had upfront unresectable or borderline resectable metastatic disease.
- Included patients in the trials received current ESMO and German S3 mCRC guideline adherent first-line treatment with CTx and TT.^4,6^
- The studies were designed as RCT.
- All outcomes of ORR, RR, PFS, and OS were reported in the results.

The following articles were excluded from the analysis:

- Any studies in which patients received non-standard of care or experimental treatments (including, but not limited to, experimental targeted therapy drugs, immunotherapy, and hepatic intra-arterial chemotherapy).
- Studies with second line, reduced dose, maintenance, disease control or palliative treatments.

### Study Selection and Data Extraction

Three of the authors (JZ, CH, and AAS) independently reviewed the titles, abstracts, and full texts to determine study eligibility for inclusion. Differences in eligibility assessments were resolved by consensus among all three authors. Data extraction was performed by two of the authors (JZ and CH) and independently verified by two other authors (AAS, JH). The following data points were extracted from each of the eligible trials: manuscript information, antineoplastic agents used (fluoropyrimidines, irinotecan, oxaliplatin, cetuximab, panitumumab, bevacizumab), number of patients included, ORR, RR (both overall and in liver-limited disease), PFS, and OS. Data on KRAS mutation status were also collected where applicable to ensure anti-epidermal growth factor (EGFR) drugs were given according to the guidelines. Data included in the final analysis reflected the intention-to-treat population of each trial.

### Qualitative Analysis

All individual trial results included in the meta-analysis were assessed for bias using the Cochrane risk-of-bias tool for randomized trials version 2 (RoB2) by two of the authors (JZ and CH).^7^ Two other authors (AAS, JH) independently reviewed the bias assessments. Any discrepancies were resolved by consensus among all four authors. The RoB2 tool was used to make judgments on five standard domains of bias (randomization process, deviation from intended intervention, missing outcome data, measurement of outcome, and result reporting) that can possibly affect the results of randomized trials. A final overall judgement of either low risk, some concerns of bias, or high risk of bias was also derived using the RoB2 tool.

### Data Synthesis and Quantitative Analysis

The statistical analysis was carried out by extracting the hazard ratios (HR) and 95% confidence intervals (95% CI) for OS and PFS as well as the percentages for ORR and RR. The HR were determined for the time-dependent variables OS and PFS with their corresponding 95% CI and displayed in forest plots. For ORR and RR, the odds ratios (OR) with their corresponding 95% CI were identified and displayed in forest plots. The HR and OR were analyzed using a random-effects model as a large heterogeneity between the individual trials was assumed. The meta-analysis was carried out with Review Manager (Rev-Man) version 5.3.5 (The Cochrane Collaboration, The Nordic Cochrane Centre, Copenhagen, Denmark).

## Results

### Search Results and Study Selection

**Figure 1:**
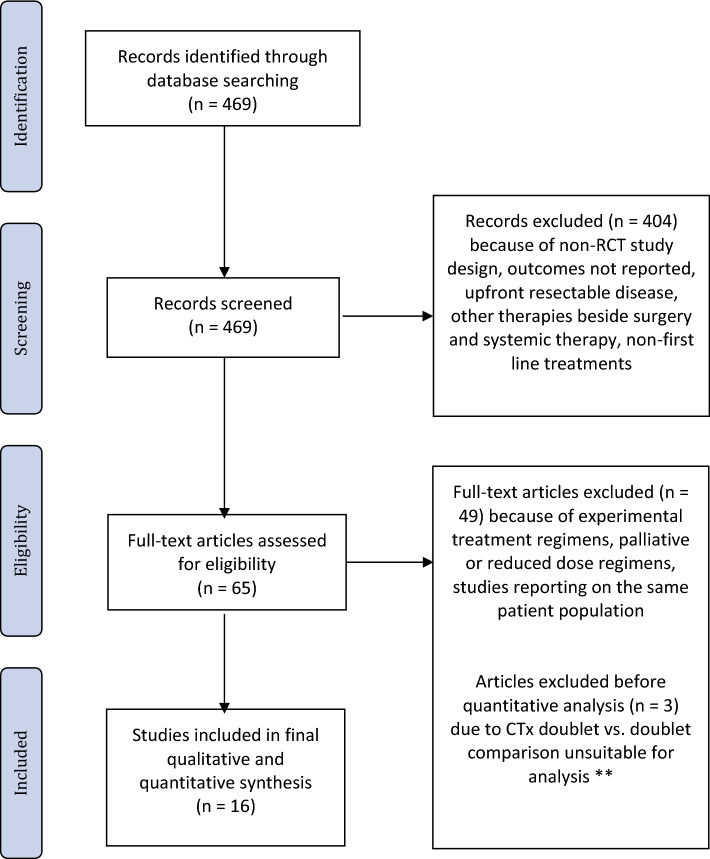
PRISMA flow diagram **^32–34^

In total, 469 records were identified from the initial database search. No duplicates were identified. After initial screening of the titles and refinement of the search strategy, two authors (JZ and AAS) assessed the full texts of 65 articles for eligibility. All the included studies used ESMO and German S3 guideline adherent treatment regimens with doublet or triplet CTx with or without anti-EGFR and anti-VEGF TT (Table 1). For studies using anti-EGFR therapies, only data for patients with KRAS wild-type tumors was included in the final analysis. Ultimately, 16 studies with 5724 patients were selected for the meta-analysis (Table 1, Figure 1).

**Table 1:** Included RCT

First author	Year	Title
Bokemeyer^8^	2011	Efficacy according to biomarker status of cetuximab plus FOLFOX-4 as first-line treatment for metastatic colorectal cancer: the OPUS study.
Cremolini^10^	2015	FOLFOXIRI plus bevacizumab versus FOLFIRI plus bevacizumab as first-line treatment of patients with metastatic colorectal cancer: updated overall survival and molecular subgroup analyses of the open-label, phase 3 TRIBE study.
Douillard^11^	2014	Final results from PRIME: randomized phase III study of panitumumab with FOLFOX4 for first-line treatment of metastatic colorectal cancer.
Falcone^12^	2007	Phase III trial of infusional fluorouracil, leucovorin, oxaliplatin, and irinotecan (FOLFOXIRI) compared with infusional fluorouracil, leucovorin, and irinotecan (FOLFIRI) as first-line treatment for metastatic colorectal cancer: the Gruppo Oncologico Nord Ovest.
Gruenberger^20^	2015	Bevacizumab plus mFOLFOX-6 or FOLFOXIRI in patients with initially unresectable liver metastases from colorectal cancer: the OLIVIA multinational randomised phase II trial.
Heinemann^23^	2014	FOLFIRI plus cetuximab versus FOLFIRI plus bevacizumab as first-line treatment for patients with metastatic colorectal cancer (FIRE-3): a randomised, open-label, phase 3 trial.
Hurwitz^13^	2019	Phase II Randomized Trial of Sequential or Concurrent FOLFOXIRI-Bevacizumab Versus FOLFOX-Bevacizumab for Metastatic Colorectal Cancer (STEAM).
Loupakis^9^	2014	Initial therapy with FOLFOXIRI and bevacizumab for metastatic colorectal cancer.
Modest^14^	2019	FOLFOXIRI Plus Panitumumab As First-Line Treatment of *RAS* Wild-Type Metastatic Colorectal Cancer: The Randomized, Open-Label, Phase II VOLFI Study (AIO KRK0109).
Passardi^15^	2015	Effectiveness of bevacizumab added to standard chemotherapy in metastatic colorectal cancer: final results for first-line treatment from the ITACa randomized clinical trial.
Rivera^16^	2017	Final analysis of the randomised PEAK trial: overall survival and tumour responses during first-line treatment with mFOLFOX6 plus either panitumumab or bevacizumab in patients with metastatic colorectal carcinoma.
Saltz^17^	2008	Bevacizumab in combination with oxaliplatin-based chemotherapy as first-line therapy in metastatic colorectal cancer: a randomized phase III study.
Souglakos^18^	2006	FOLFOXIRI (folinic acid, 5-fluorouracil, oxaliplatin and irinotecan) versus FOLFIRI (folinic acid, 5-fluorouracil and irinotecan) as first-line treatment in metastatic colorectal cancer (MCC): a multicentre randomised phase III trial from the Hellenic Oncology Research Group (HORG).
Van Cutsem^19^	2011	Cetuximab plus irinotecan, fluorouracil, and leucovorin as first-line treatment for metastatic colorectal cancer: updated analysis of overall survival according to tumor KRAS and BRAF mutation status.
Ychou^21^	2013	A randomized phase II trial of three intensified chemotherapy regimens in first-line treatment of colorectal cancer patients with initially unresectable or not optimally resectable liver metastases. The METHEP trial.
Ye^22^	2013	Randomized controlled trial of cetuximab plus chemotherapy for patients with KRAS wild-type unresectable colorectal liver-limited metastases.

### Qualitative Analysis

From the included studies, 16 primary and 35 secondary outcomes were selected for risk of bias assessments with RoB2. A visual presentation of the bias assessments is presented in Supplementary Table 1. Three of the included primary outcomes were judged to be at a low risk for bias, 12 were judged to have some concern for bias, and one was judged to be at a high risk of bias. The main areas of possible concern were the randomization process and the measurement of the outcomes. Specifically, most studies did not report whether the allocation sequence was concealed until participants were enrolled and assigned to the interventions. Another area of possible concern was the frequent omission of blinded and centralized radiological review which could affect ORR and PFS results. An overall judgment of high risk of bias was made for one of the primary outcomes due to the baseline differences between groups, suggesting a possible problem with the randomization process and possible selection of the reported results. For the 35 secondary outcomes, a similar distribution of bias risk was observed (Supplementary Table 1).

## Quantitative Analysis

### General Study Characteristics

Thirteen trials included mCRC with single or multiple sites that were primarily unresectable or borderline.^8–19^ Three trials included patients with liver-limited disease.^20–22^ The range of liver-only metastasis was between 18 and 33% for the studies, including patients with multiple sites.

### Treatment Approaches

Seven trials with 3505 patients compared doublet (FOLFOX, FOLFIRI, XELOX) or triplet (FOLFOXIRI) CTx + TT (cetuximab, panitumumab, bevacizumab) versus doublet or triplet CTx only.^8,11,14,15,17,19,22^ Four trials with 868 patients compared triplet CTx + TT versus doublet CTx + TT.^9,10,13,20^ Three trials with 589 patients compared triplet CTx versus doublet CTx only.^12,18,21^ Two studies with 762 patients compared doublet CTx + anti-EGFR TT (cetuximab, panitumumab) versus doublet CTx + anti-VEGF TT (bevacizumab).^16,23^

### Overall Response Rates (ORR)

**Figure 2:**
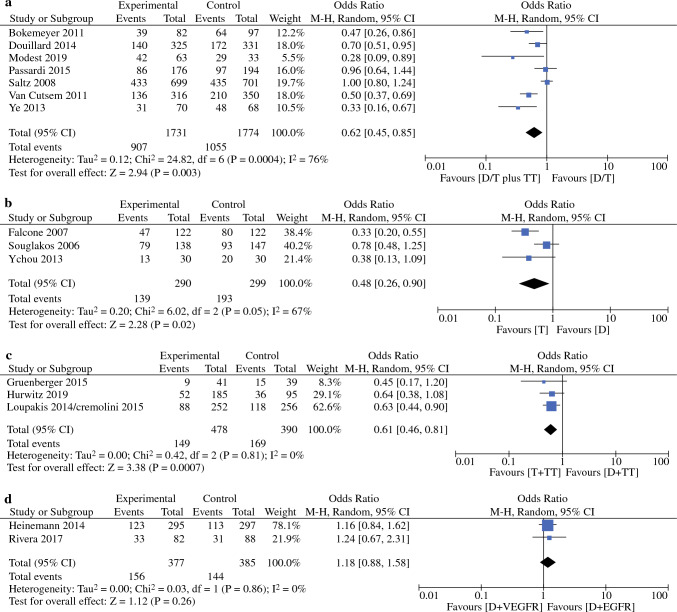
**a** Overall response rate of studies comparing doublet or triplet chemotherapy with targeted therapy versus doublet or triplet chemotherapy in patients with stage IV colorectal cancer. **b** Overall response rate of studies comparing doublet versus triplet chemotherapy in patients with stage IV colorectal cancer.

The ORR ranged from 29 to 87% (average 54%, median 57%). The comparisons of doublet/triplet CTx + TT versus doublet/triplet CTx only (OR: 0.62 [95 CI: 0.45; 0.85]; *p* = 0.003); Fig. 2a,^8,11,14,15,17,19^ triplet CTx versus doublet CTx only (OR: 0.48 [95% CI: 0.26; 0.90]; *p* = 0.02); Fig. 2b,^11,22^ and triplet CTx + TT versus doublet CTx + TT (OR: 0.61 [95% CI 0.46; 0.81]; *p* < 0.001); Fig. 2c^9,10,13,20^ were associated with statistically significant differences in ORR. No differences in ORR were observed regarding the type of TT given (anti-EGFR versus anti-VEGF) (OR: 1.18 [95% CI 0.88; 1.58]; *p* = 0.26); Fig. 2d.^16,23^

### Resection Rates (RR)

**Figure 3:**
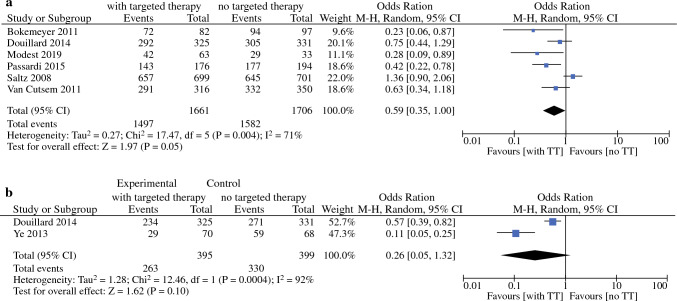
**a** Resection rate of studies comparing doublet or triplet chemotherapy with targeted therapy versus doublet/triplet chemotherapy in patients with stage IV colorectal cancer. **b** Resection rate in liver-only colorectal liver metastases of studies comparing doublet or triplet chemotherapy with targeted therapy versus doublet or triplet chemotherapy in patients with stage IV colorectal cancer.

Only three of the included RCT reported detailed criteria for metastasis resectability and how the decision for resection was made by the multidisciplinary team.^20–22^ The overall RR ranged from 3 to 33% (average and median both 12%; Supplementary Table 2). The overall RR was better in patients that received CTx + TT versus CTx only; Fig. 3a.^8,11,14,15,17,19^ The RR in patients with liver-limited disease ranged from 13 to 67% (average 29%, median 35%). However, no difference in RR for patients with liver-limited disease treated with doublet CTx + TT versus doublet CTx only was observed; Fig. 3b.^11,22^

### Progression-Free Survival (PFS)

PFS was better in patients treated with CTx + TT versus CTx only (OR: 0.79 [95% CI 0.72; 0.87]; *p* < 0.001) and in patients treated with triplet CTx + TT versus doublet CTx + TT (OR: 0.68 [95% CI 0.50; 0.90]; *p* = 0.009). No differences in PFS were observed for studies which compared doublet versus triplet CTx only (OR: 0.79 [95% CI 0.50; 1.23]; *p* = 0.30), and CTx + anti-EGFR TT versus CTx + anti-VEGF TT (OR: 0.82 [95% CI 0.61; 1.11]; *p* = 0.19). Data are displayed in Supplementary Figs. 1a to d.

### Overall Survival (OS)

OS was better in patients treated with CTx + TT versus CTx only (OR: 0.84 [95% CI 0.74; 0.95]; *p* = 0.007), patients treated with triplet CTx + TT versus doublet CTx + TT (OR: 0.74 [95% CI 0.55; 0.98]; *p* = 0.04), and in patients treated with doublet CTx + anti-EGFR TT versus doublet CTx + anti-VEGF TT (OR: 0.70 [95% CI 0.55; 0.89]; *p* = 0.003). No differences in OS were observed for patients treated with triplet versus doublet CTx only (OR: 0.89 [95% CI 0.75; 1.05]; *p* = 0.17). Data are displayed in Supplementary Figs. 2a to d.

## Discussion

This meta-analysis explored how modern systemic therapy affects ORR and RR in patients with upfront unresectable or borderline resectable mCRC. More than 50% of included patients had an objective response to the treatment; however, the overall RR were much lower as they almost never exceeded 15%. RR were higher (around 30%) in patients with liver-limited disease. Both ORR and RR were higher in patients who were treated with both CTx and TT.

Previously published RR for oligometastatic disease range from 5 to 22%. The overall RR in this meta-analysis matches the previously published oligometastatic RR; however, patients with liver-limited disease have higher RR. Few RCT have reported RR for liver-limited mCRC. One of them is the CELIM trial published by Folprecht et al. in 2010 which compared treatment with cetuximab and FOLFOX6 versus cetuximab and FOLFIRI in patients with unresectable colorectal liver metastases. The CELIM trial was not included in this analysis as it did not compare regimens with TT versus regimens without TT. Folprecht et al. reported an ORR of 62%. This was similar to the ORR for patients treated with doublet CTx and anti-EGFR TT in this meta-analysis (Supplementary Table 2). Folprecht et al. reported an R0 RR of 34% which is again similar to observed RR for liver-limited disease in our meta-analysis. More interestingly, Folprecht et al. also reported the results of a blinded post-treatment surgical review of resectability using CT and MRI scans. The post-treatment review involved seven senior hepatobiliary surgeons. They judged that 60% of the patients included in the CELIM trial could have undergone potentially curative resection after systemic treatment. This represents a major potential increase in resectability.^24^ Modest et al. also published a similar blinded post-treatment review of 448 patients included in the FIRE-3 trial.^23,25^ In the review by Modest et al, eight experienced surgeons and three medical oncologists centrally reviewed CT and MRI examinations from the FIRE-3 trial. Included patients were considered resectable if at least 50% of the reviewers selected surgical intervention. Surgical intervention was retrospectively considered possible in 22% of patients at baseline and 53% at best response. However, the actual resection rate achieved in the FIRE-3 trial was 13%. Again, the discrepancy between potential and actual resection rates demonstrates the necessary impact of expert involvement in determining resectability of mCRC.^20,25^ The recently published CAIRO05 trial adds new evidence for the strategy in removing metastases from the liver and thus prolonging tumor-free intervals, with a positive impact on overall survival. In this trial an expert panel evaluated the tumors before chemotherapy and judged that approximately 82% to 88% may become resectable (or treatable with ablation). After the chemotherapy, patients with mutations in either RAS or BRAF underwent ablation (37% of patients) or surgery (51% of patients), whereas patients with RAS/BRAF wild-type underwent complete local treatment with either surgery and/or ablation in 68% (RAS) and 69% (BRAF) of the cases.^26^ This finally implements the findings of the CLOCC trial into a common approach and equipoise of resection and ablation in a high-quality clinical trial.^3^

The current meta-analysis has several limitations. One is risk of inclusion bias due to missed publications or mistakes in determining whether a study was eligible for inclusion in this meta-analysis. However, this specific risk was minimized by having three of the authors perform the literature search and study selection independently and strictly defining the inclusion and exclusion criteria. In judging whether studies used guideline-adherent treatments we followed the ESMO guidelines which are considered the gold standard for treatment of mCRC.^4,27^ However, most included publications shaped the guidelines over time and were the basis for improved treatment strategies. One other important limitation is that the location of the primary tumor or biomarkers other than KRAS were not considered in this meta-analysis. There is accumulating evidence in the literature that the location and biomarker status of both the primary tumor and metastases play a significant role in choosing the right systemic treatment with CTx, TT, or immunotherapy.^4,6,28–30^ However, with the currently available RCT data, further subgroup analyses were not possible. The meta-analysis also has several strengths. Only RCT were included and the guidelines published in the Cochrane Handbook, which is considered the gold standard for systematic reviews, were followed.^7,31^ The standardized qualitative assessments of the included RCT revealed a predominantly low to moderate risk of bias. The included 16 RCT comprised 5724 patients which provided a large sample size for quantitative analysis.

In conclusion, the evolution of the treatment of mCRC has improved outcomes over the last 20 years. Nonetheless, there is still potential for improving treatment decisions regarding resection of mCRC. Our meta-analysis has shown significant variability in RR which is consistent with previously published findings. Furthermore, few RCT reported how decisions regarding resectability were made. In our opinion, this strongly advocates for the indispensable involvement of surgeons experienced in the management of mCRC and borderline resectable tumors in all multidisciplinary teams. The exact composition and qualifications of the members of the multidisciplinary treatment team should receive special attention in the design of future RCT to improve RR, which has been shown to improve overall survival significantly.^5^ There will probably never be a sufficient answer to what resectability or unresectability is, as this question requires multiple considerations for each individual patient. However, the toolbox definitely must include the following: an experienced liver surgeon; an interdisciplinary discussion based on oncologic principles, including mutational status; a risk-benefit evaluation of patient factors and patient will; and a repeat discussion after every restaging and change of therapies.

### Supplementary Information

Below is the link to the electronic supplementary material.

Supplementary file1 (PPTX 1322 kb)

Supplementary file2 (DOCX 15 kb)

Supplementary file3 (XLSX 20 kb)

## References

[1] Filmann N, Walter D, Schadde E, et al. Mortality after liver surgery in Germany. *Br J Surg.* Published Online First: 24 July 2019. doi:10.1002/bjs.11236 10.1002/bjs.11236 31339558

[2] Creasy JM, Sadot E, Koerkamp BG (2018). Actual 10-year survival after hepatic resection of colorectal liver metastases: what factors preclude cure?. Surgery..

[3] Ruers T, Van Coevorden F, Punt CJA (2017). Local treatment of unresectable colorectal liver metastases: results of a randomized phase II Trial. J Natl Cancer Inst.

[4] Van Cutsem E, Cervantes A, Adam R (2016). ESMO consensus guidelines for the management of patients with metastatic colorectal cancer. Ann Oncol.

[5] Zeineddine FA, Zeineddine MA, Yousef A (2023). Survival improvement for patients with metastatic colorectal cancer over twenty years. Npj Precis Oncol.

[6] Leitlinienprogramm Onkologie (Deutsche Krebsgesellschaft, Deutsche Krebshilfe, AWMF): S3-Leitlinie Kolorektales Karzinom, Langversion 2.1, 2019, AWMF Registrierungsnummer: 021/007OL, http://www.leitlinienprogramm- onkologie.de/leitlinien/kolorektales-karzinom/.

[7] Higgins JPT, Thomas J, Chandler J, Cumpston M, Li T, Page MJ, Welch VA, editors. Cochrane handbook for systematic reviews of interventions, version 6.4. Cochrane; 2023.

[8] Bokemeyer C, Bondarenko I, Hartmann JT (2011). Efficacy according to biomarker status of cetuximab plus FOLFOX-4 as first-line treatment for metastatic colorectal cancer: the OPUS study. Ann Oncol Off J Eur Soc Med Oncol.

[9] Loupakis F, Cremolini C, Masi G (2014). Initial therapy with FOLFOXIRI and bevacizumab for metastatic colorectal cancer. N Engl J Med.

[10] Cremolini C, Loupakis F, Antoniotti C (2015). FOLFOXIRI plus bevacizumab versus FOLFIRI plus bevacizumab as first-line treatment of patients with metastatic colorectal cancer: updated overall survival and molecular subgroup analyses of the open-label, phase 3 TRIBE study. Lancet Oncol.

[11] Douillard JY, Siena S, Cassidy J (2014). Final results from PRIME: randomized phase III study of panitumumab with FOLFOX4 for first-line treatment of metastatic colorectal cancer. Ann Oncol Off J Eur Soc Med Oncol.

[12] Falcone A, Ricci S, Brunetti I (2007). Phase III trial of infusional fluorouracil, leucovorin, oxaliplatin, and irinotecan (FOLFOXIRI) compared with infusional fluorouracil, leucovorin, and irinotecan (FOLFIRI) as first-line treatment for metastatic colorectal cancer: the Gruppo Oncologico Nord Ovest. J Clin Oncol Off J Am Soc Clin Oncol.

[13] Hurwitz HI, Tan BR, Reeves JA (2019). Phase II randomized trial of sequential or concurrent FOLFOXIRI-bevacizumab versus FOLFOX-bevacizumab for metastatic colorectal cancer (STEAM). Oncologist.

[14] Modest DP, Martens UM, Riera-Knorrenschild J (2019). FOLFOXIRI plus panitumumab as first-line treatment of RAS Wild-type metastatic colorectal cancer: the randomized, open-label, phase II VOLFI Study (AIO KRK0109). J Clin Oncol Off J Am Soc Clin Oncol.

[15] Passardi A, Nanni O, Tassinari D (2015). Effectiveness of bevacizumab added to standard chemotherapy in metastatic colorectal cancer: final results for first-line treatment from the ITACa randomized clinical trial. Ann Oncol Off J Eur Soc Med Oncol.

[16] Rivera F, Karthaus M, Hecht JR (2017). Final analysis of the randomised PEAK trial: overall survival and tumour responses during first-line treatment with mFOLFOX6 plus either panitumumab or bevacizumab in patients with metastatic colorectal carcinoma. Int J Colorectal Dis.

[17] Saltz LB, Clarke S, Díaz-Rubio E (2008). Bevacizumab in combination with oxaliplatin-based chemotherapy as first-line therapy in metastatic colorectal cancer: a randomized phase III study. J Clin Oncol Off J Am Soc Clin Oncol.

[18] Souglakos J, Androulakis N, Syrigos K (2006). FOLFOXIRI (folinic acid, 5-fluorouracil, oxaliplatin and irinotecan) versus FOLFIRI (folinic acid, 5-fluorouracil and irinotecan) as first-line treatment in metastatic colorectal cancer (MCC): a multicentre randomised phase III trial from the hellenic oncology research group (HORG). Br J Cancer.

[19] Van Cutsem E, Köhne C-H, Láng I (2011). Cetuximab plus irinotecan, fluorouracil, and leucovorin as first-line treatment for metastatic colorectal cancer: updated analysis of overall survival according to tumor KRAS and BRAF mutation status. J Clin Oncol Off J Am Soc Clin Oncol.

[20] Gruenberger T, Bridgewater J, Chau I (2015). Bevacizumab plus mFOLFOX-6 or FOLFOXIRI in patients with initially unresectable liver metastases from colorectal cancer: the OLIVIA multinational randomised phase II trial. Ann Oncol Off J Eur Soc Med Oncol.

[21] Ychou M, Rivoire M, Thezenas S (2013). A randomized phase II trial of three intensified chemotherapy regimens in first-line treatment of colorectal cancer patients with initially unresectable or not optimally resectable liver metastases The METHEP trial. Ann Surg Oncol.

[22] Ye L-C, Liu T-S, Ren L (2013). Randomized controlled trial of cetuximab plus chemotherapy for patients with KRAS wild-type unresectable colorectal liver-limited metastases. J Clin Oncol Off J Am Soc Clin Oncol.

[23] Heinemann V, von Weikersthal LF, Decker T (2014). FOLFIRI plus cetuximab versus FOLFIRI plus bevacizumab as first-line treatment for patients with metastatic colorectal cancer (FIRE-3): a randomised, open-label, phase 3 trial. Lancet Oncol.

[24] Folprecht G, Gruenberger T, Bechstein W (2014). Survival of patients with initially unresectable colorectal liver metastases treated with FOLFOX/cetuximab or FOLFIRI/cetuximab in a multidisciplinary concept (CELIM-study). Ann Oncol Off J Eur Soc Med Oncol ESMO.

[25] Modest DP, Denecke T, Pratschke J (1990). Surgical treatment options following chemotherapy plus cetuximab or bevacizumab in metastatic colorectal cancer—central evaluation of FIRE-3. Eur J Cancer Oxf Engl.

[26] Bond MJG, Bolhuis K, Loosveld OJL (2023). First-line systemic treatment strategies in patients with initially unresectable colorectal cancer liver metastases (CAIRO5): an open-label, multicentre, randomised, controlled, phase 3 study from the Dutch Colorectal Cancer Group. Lancet Oncol.

[27] Cervantes A, Adam R, Roselló S (2023). Metastatic colorectal cancer: ESMO clinical practice guideline for diagnosis, treatment and follow-up. Ann Oncol Off J Eur Soc Med Oncol.

[28] Guinney J, Dienstmann R, Wang X (2015). The consensus molecular subtypes of colorectal cancer. Nat Med.

[29] Pitroda SP, Khodarev NN, Huang L (2018). Integrated molecular subtyping defines a curable oligometastatic state in colorectal liver metastasis. Nat Commun.

[30] Martini G, Dienstmann R, Ros J (2020). Molecular subtypes and the evolution of treatment management in metastatic colorectal cancer. Ther Adv Med Oncol.

[31] Howick J, Chalmers I, Glasziou P, Greenhalgh T, Heneghan C, Liberati A, et al. Explanation of the 2011 Oxford Centre for Evidence-Based Medicine (OCEBM) Levels of Evidence (Background Document). Oxford Centre for Evidence-Based Medicine. https://www.cebm.ox.ac.uk/resources/levels-of-evidence/ocebmlevels-of-evidence.

[32] Carrato A, Abad A, Massuti B (1990). First-line panitumumab plus FOLFOX4 or FOLFIRI in colorectal cancer with multiple or unresectable liver metastases: a randomised, phase II trial (PLANET-TTD). Eur J Cancer Oxf Engl..

[33] Ducreux M, Bennouna J, Hebbar M (2011). Capecitabine plus oxaliplatin (XELOX) versus 5-fluorouracil/leucovorin plus oxaliplatin (FOLFOX-6) as first-line treatment for metastatic colorectal cancer. Int J Cancer..

[34] Yamazaki K, Nagase M, Tamagawa H (2016). Randomized phase III study of bevacizumab plus FOLFIRI and bevacizumab plus mFOLFOX6 as first-line treatment for patients with metastatic colorectal cancer (WJOG4407G). Ann Oncol Off J Eur Soc Med Oncol.

