# Supplementary material for: Chemotherapy and Targeted Therapy Strategies in Patients with Unresectable or Borderline Resectable Metastatic Colorectal Cancer: Evidence for a Lack of Focus on Resection Rates
Source: Ann Surg Oncol. 2023 Aug 30;30(12):7624–32. doi: 10.1245/s10434-023-14049-3 (PMC10562287; doi:10.1245/s10434-023-14049-3)
Supplement: Supplementary file 1 — Supplementary file1 (PPTX 1322 kb) [file 10434_2023_14049_MOESM1_ESM.pptx]

## Slide 1
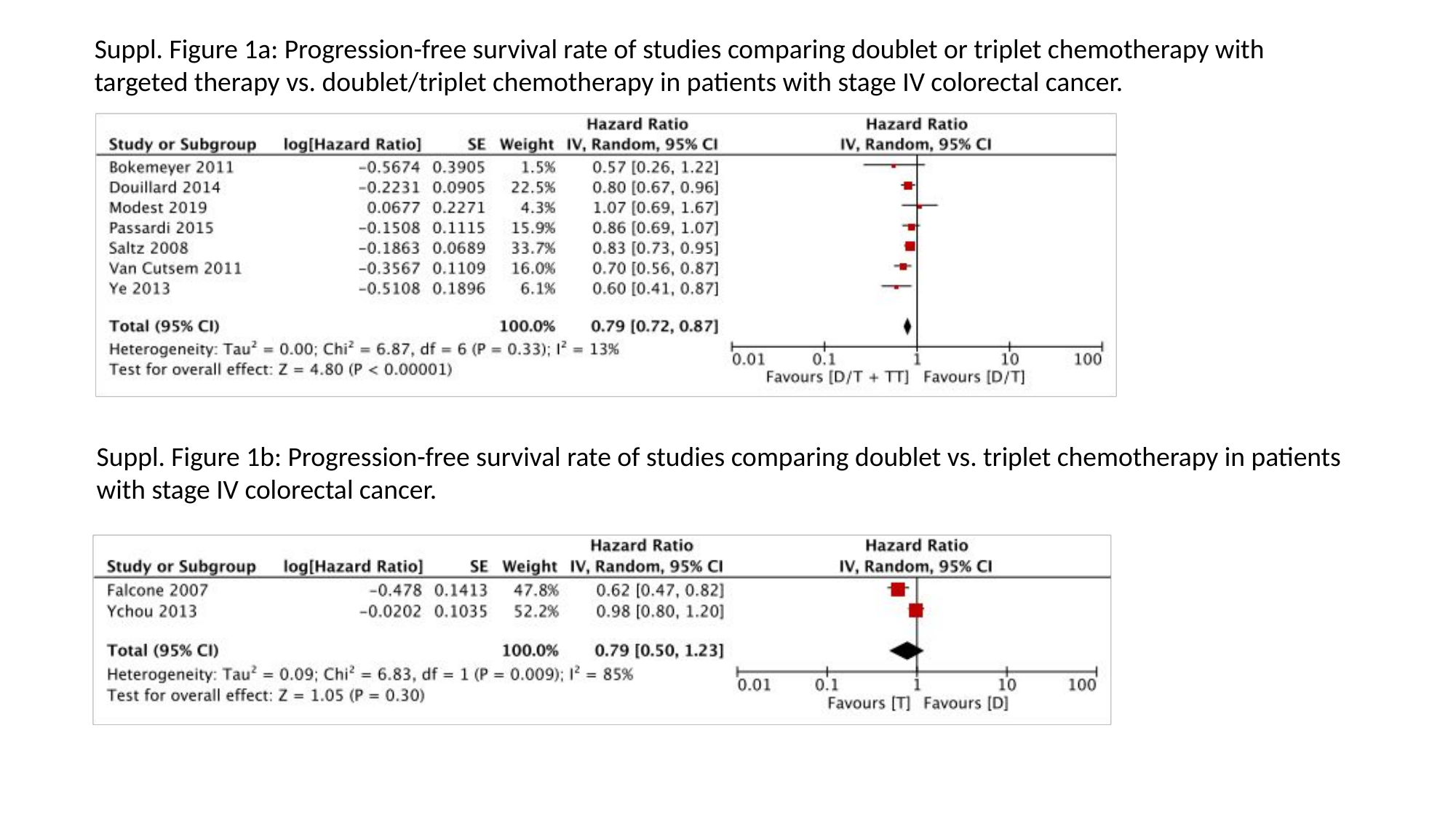

Suppl. Figure 1a: Progression-free survival rate of studies comparing doublet or triplet chemotherapy with targeted therapy vs. doublet/triplet chemotherapy in patients with stage IV colorectal cancer.
Suppl. Figure 1b: Progression-free survival rate of studies comparing doublet vs. triplet chemotherapy in patients with stage IV colorectal cancer.

## Slide 2
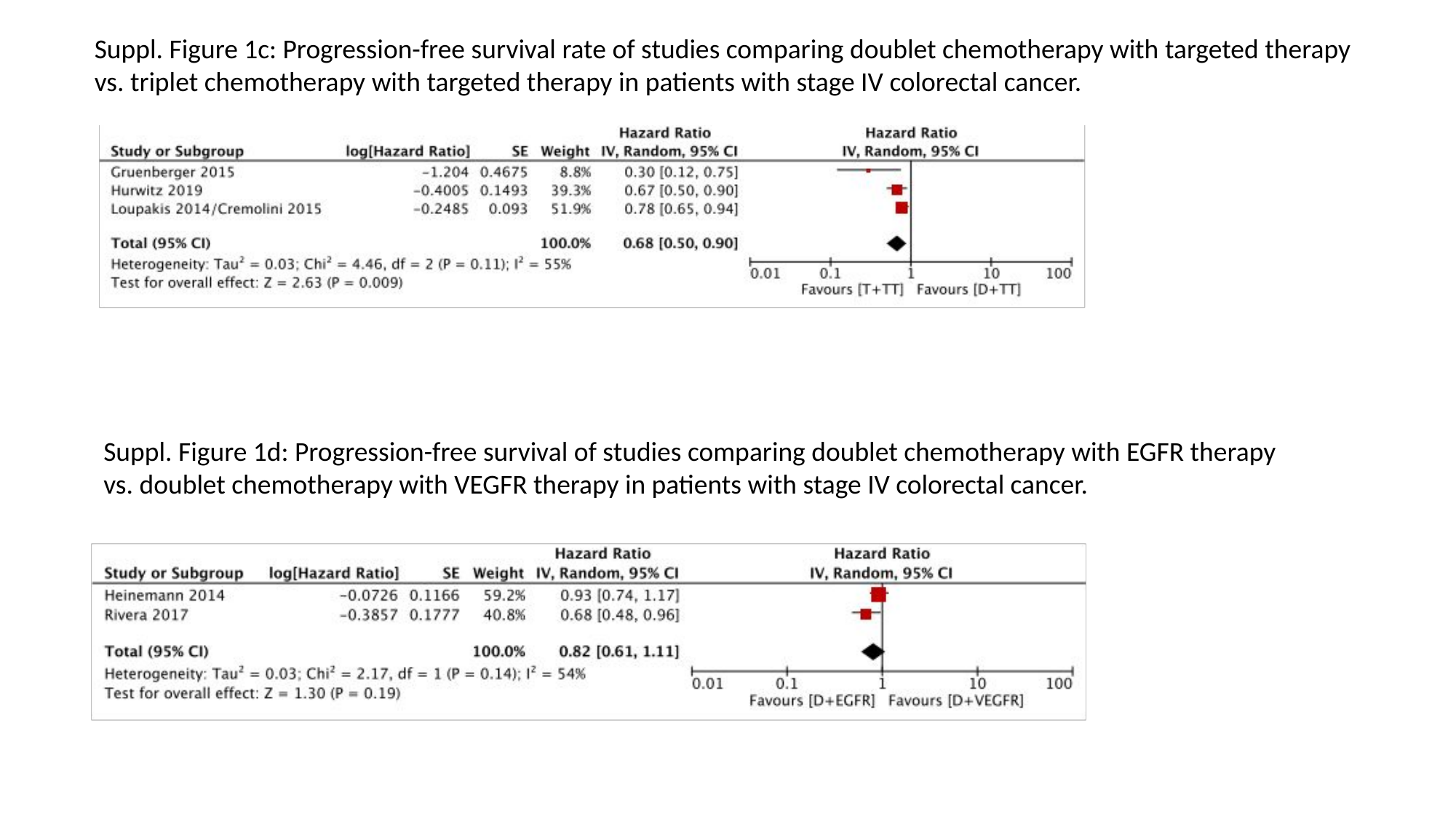

Suppl. Figure 1c: Progression-free survival rate of studies comparing doublet chemotherapy with targeted therapy
vs. triplet chemotherapy with targeted therapy in patients with stage IV colorectal cancer.
Suppl. Figure 1d: Progression-free survival of studies comparing doublet chemotherapy with EGFR therapy
vs. doublet chemotherapy with VEGFR therapy in patients with stage IV colorectal cancer.

## Slide 3
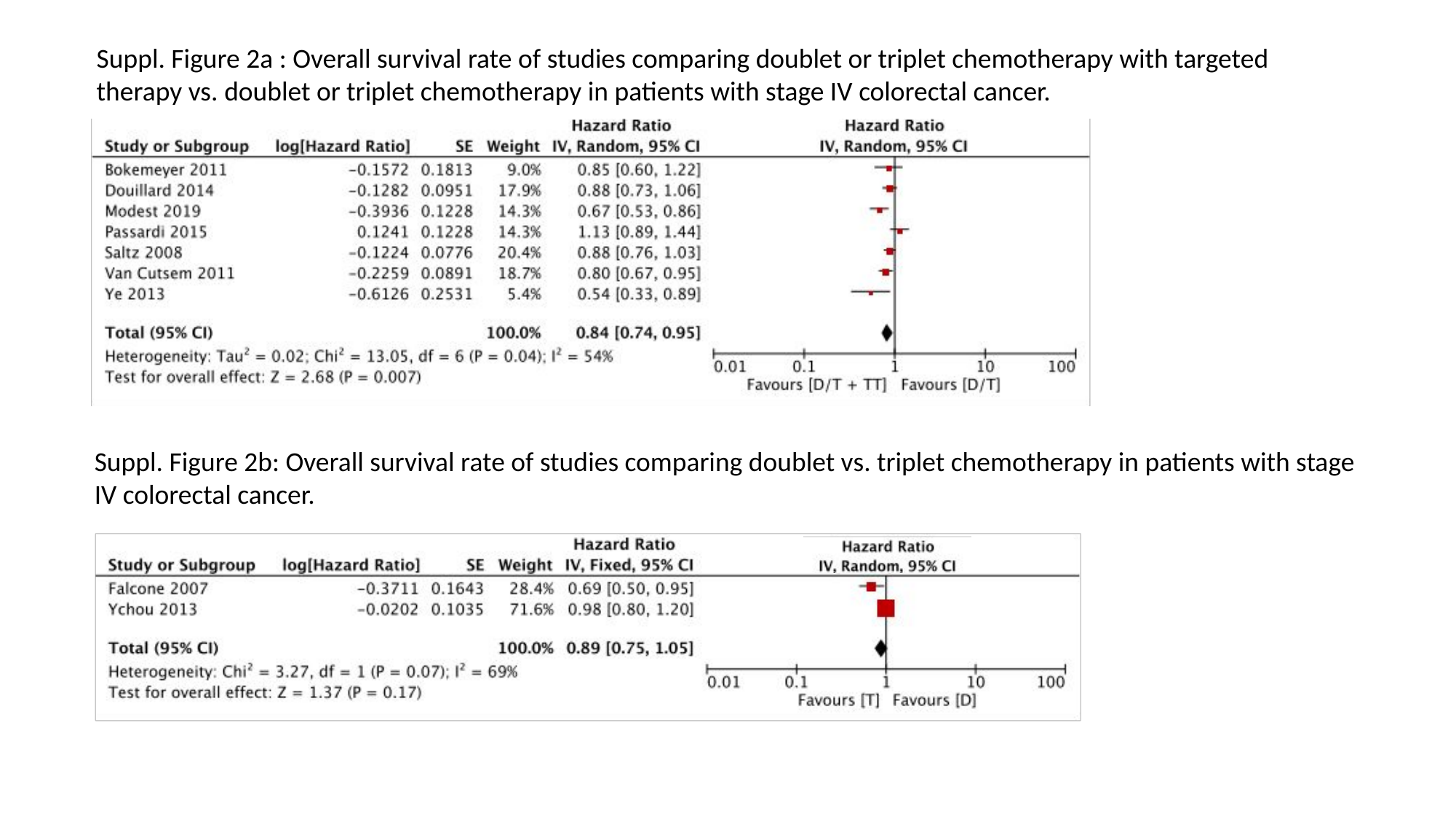

Suppl. Figure 2a : Overall survival rate of studies comparing doublet or triplet chemotherapy with targeted therapy vs. doublet or triplet chemotherapy in patients with stage IV colorectal cancer.
Suppl. Figure 2b: Overall survival rate of studies comparing doublet vs. triplet chemotherapy in patients with stage IV colorectal cancer.

## Slide 4
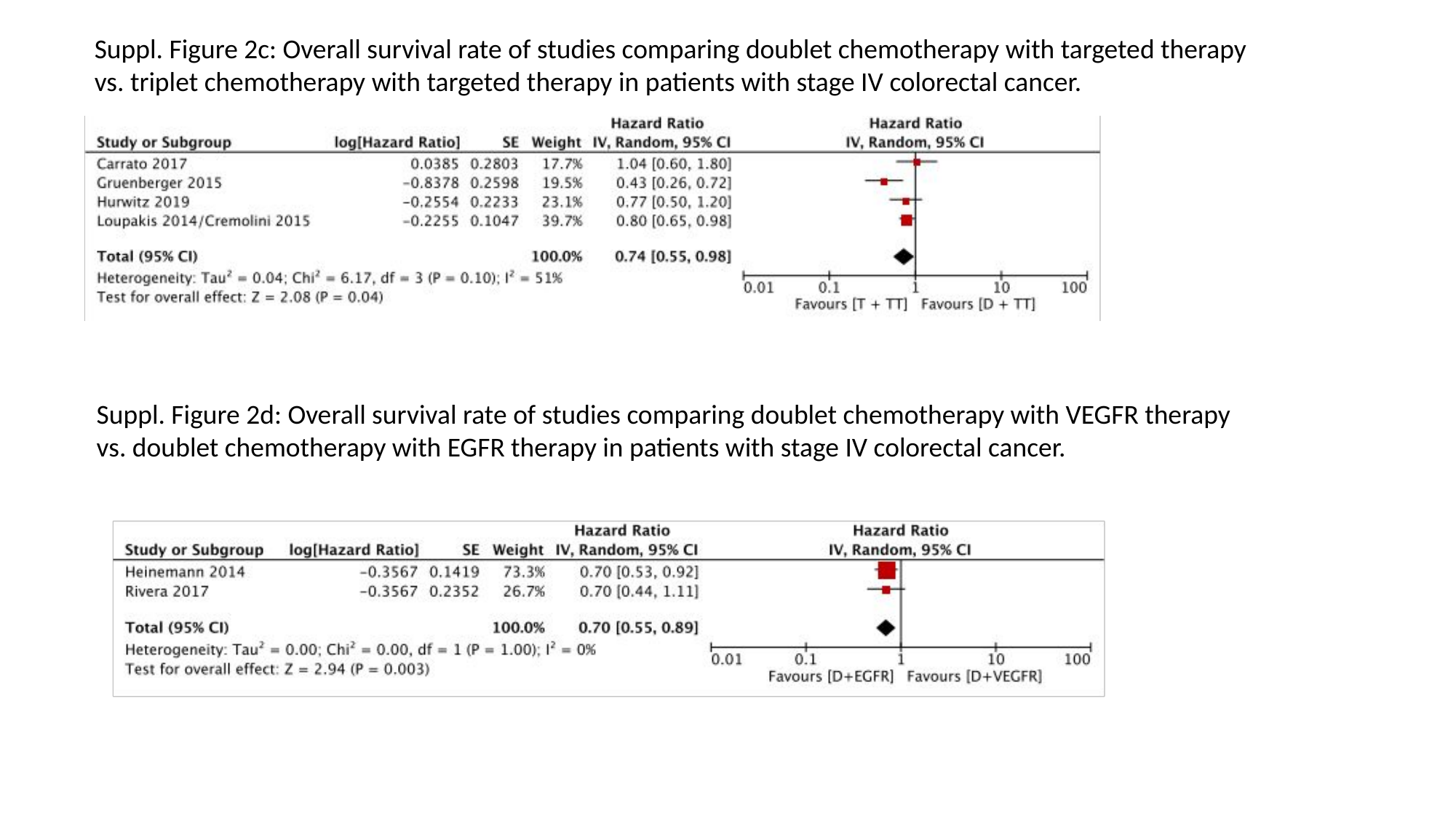

Suppl. Figure 2c: Overall survival rate of studies comparing doublet chemotherapy with targeted therapy
vs. triplet chemotherapy with targeted therapy in patients with stage IV colorectal cancer.
Suppl. Figure 2d: Overall survival rate of studies comparing doublet chemotherapy with VEGFR therapy
vs. doublet chemotherapy with EGFR therapy in patients with stage IV colorectal cancer.
