# Supplementary material for: Chemotherapy and Targeted Therapy Strategies in Patients with Unresectable or Borderline Resectable Metastatic Colorectal Cancer: Evidence for a Lack of Focus on Resection Rates
Source: Ann Surg Oncol. 2023 Aug 30;30(12):7624–32. doi: 10.1245/s10434-023-14049-3 (PMC10562287; doi:10.1245/s10434-023-14049-3)
Supplement: Supplementary file 2 — Supplementary file2 (DOCX 15 kb) [file 10434_2023_14049_MOESM2_ESM.docx]

| **Bias assessments for primary endpoints** | | | | | | | | | **Overall bias for secondary endpoints** | | | |
| --- | --- | --- | --- | --- | --- | --- | --- | --- | --- | --- | --- | --- |
| **First author** | **Year** | **Primary endpoint** | **Randomization process** | **Deviations from intended interventions** | **Missing outcome data** | **Measurement of the outcome** | **Selection of the reported result** | **Overall** | **OS** | **PFS** | **ORR** | **RR** |
| Bokemeyer | 2011 | ORR |  |  |  |  |  |  |  |  | NA | NA |
| Cremolini | 2015 | PFS |  |  |  |  |  |  |  | NA |  | NA |
| Douillard | 2014 | PFS |  |  |  |  |  |  |  | NA | NA | NA |
| Falcone | 2007 | RR |  |  |  |  |  |  |  |  | NA | NA |
| Gruenberger | 2015 | RR |  |  |  |  |  |  | NA |  |  | NA |
| Heinemann | 2014 | objective response |  |  |  |  |  |  |  | NA | NA | NA |
| Hurwitz | 2019 | ORR |  |  |  |  |  |  |  |  | NA |  |
| Loupakis | 2014 | PFS |  |  |  |  |  |  |  | NA |  |  |
| Modest | 2019 | ORR |  |  |  |  |  |  |  |  | NA |  |
| Passardi | 2015 | PFS |  |  |  |  |  |  |  | NA |  | NA |
| Rivera | 2017 | PFS |  |  |  |  |  |  |  | NA |  | NA |
| Saltz | 2008 | PFS |  |  |  |  |  |  |  | NA | NA |  |
| Souglakos | 2016 | OS |  |  |  |  |  |  | NA |  | NA |  |
| Van Cutsem | 2011 | PFS |  |  |  |  |  |  |  | NA |  | NA |
| Ychou | 2013 | ORR |  |  |  |  |  |  |  |  | NA |  |
| Ye | 2013 | conversion to resection |  |  |  |  |  |  |  |  |  | NA |

| Low risk |  |
| --- | --- |
| Some concerns |  |
| High risk |  |
| NA | not available |

Supplementary table 1. RoB2 bias assessments.
